# Human papillomavirus infection a favorable prognostic factor in laryngeal squamous cell carcinoma is associated with the expression of proliferating cell nuclear antigen

**DOI:** 10.12669/pjms.295.3606

**Published:** 2013

**Authors:** Hua Jiang, Peng-Fang Lin

**Affiliations:** 1 **Hua Jiang, MD, PhD, Department of Otolaryngology, 2nd Affiliated Hospital, College of Medicine, Zhejiang University, Zhejiang, China.**; 2 **Peng-Fang Lin, MD, Department of Otolaryngology, 2nd Affiliated Hospital, College of Medicine, Zhejiang University, Zhejiang, China.**

**Keywords:** Human papillomavirus, Proliferating cell nuclear antigen, Laryngeal squamous cell carcinoma, Prognosis

## Abstract

***Objective:*** It has been documented that human papilloma virus (HPV) DNA replication requires proliferating cell nuclear antigen (PCNA). However the association between them in tumors is still controversial. Up to now, the role of HPV in laryngeal squamous cell carcinoma (LSCC) has not been clearly established, and the correlation between HPV and PCNA in LSCC remains poorly explored.

***Methods:*** We retrospectively reviewed the clinicopathological features and follow-up data of 71 patients with LSCC. The lesions were examined for PCNA using immunohistochemistry, and for HPV using in situ hybridization.

***Results:*** 31 (43.7%) cases showed infection of HPV and 38 (53.5%) showed overexpression of PCNA. No significant difference of HPV status in clinicopathological features was found. While there was a significant difference of PCNA expression in histology grade but no significant difference of PCNA expression in other clinicopathological features could be detected, and the expression of PCNA is not a significant predictor of survival in LSCC patients. However, HPV infection is a favorable prognostic factor in LSCC patients. Moreover, HPV infection is associated with PCNA overexpression.

***Conclusion:*** Human papilloma virus (HPV) infection is an indicator of better prognosis in LSCC and associated with the expression of PCNA.

## INTRODUCTION

Laryngeal squamous cell carcinoma (LSCC) is one of the most common forms of head and neck cancer. The incidence is increasing continuously. Since three decades ago, human papillomavirus (HPV) has been considered as a causative agent and linked to LSCC.^1^ HPV is a small circular DNA virus, and it has been well documented that HPV is an important carcinogen in cervical cancer and over 90% of these tumors are infected with HPV. But to date the correlation between HPV and LSCC is still disputed. Many studies have reported that HPV infection is associated with a better prognosis in patients with head and neck squamous cell carcinoma (HNSCC).^2^^,^^3^ However, there are frequent controversial and contradictory results.^4^

The proliferating cell nuclear antigen (PCNA) is a nuclear polypeptide essential for DNA synthesis and replication of mammalian cells. Its expression reaches a maximum point in the S-phase of the cell cycle. The level of PCNA is one of the methods to define the tumor proliferation. PCNA has been evaluated as an unfavorable prognostic factor and might be associated with radiosensitivity.^5^ The association has been shown between PCNA expression and cell proliferation in head and neck tumors.^6^

It has been documented that HPV DNA replication requires the presence of PCNA.^7^ Some studies have investigated the correlation between the PCNA expression and HPV infection. However, the results remain highly controversial.^8^^,^^9^ To date, the relationship between the expression of PCNA and the presence of HPV in patients with LSCC remains poorly explored. In the present study, we evaluated the PCNA, HPV and clinicopathological features in the patients suffering from LSCC, and investigated the relationships between them.

## METHODS

This study consisted of 71 patients with LSCC who were diagnosed and treated with surgical management in our department between 1997 and 2008. Adjuvant radiotherapy was given in addition to surgical treatment to 39 patients. To assess the advancement of the disease, the TNM classification according to 2002 UICC was used. A minimum of two years follow-up was required, unless the patient died from the disease.

***Immunohistochemistry:*** All resection specimens were fixed in 10% formalin and rountinely processed for paraffin embedding. Five microns thick sections from the tissue blocks were placed on coated glass slides, and for immunohistochemical examination the streptavidin peroxidase method was used. The sections were deparaffinized and rehydrated, and then microwaved for an antigen retrieval procedure. Tissue sections were immunostained applying the antibody PCNA (PC-10, DAKO) in a dilution 1:100. Counterstaining was performed with hematoxylin. In negative controls the primary antibody was omitted, and a section previously shown to be positive was used as a positive control. The expression of PCNA was assessed in the cell nuclei and evaluated using a five-point grading system. Less than 10% tumor cells positive was scored 0, 10% to 25% scored as 1+, 25% to 50% scored as 2+, 50% to 75% scored as 3+, and more than 75% scored as 4+. At least 500 cells per high-power (objective lens×40) field and three fields were observed, and grades of 3+ and 4+ were regarded as overexpression.

***In Situ Hybridization (ISH):*** In situ hybridization was performed according to the protocol of HPV In Situ Hybridization/Detection Kit, (Types 16/18) (Maxim Biotech, California USA). In brief, the tissue sections (5 μm) were baked at 73℃ overnight. After deparaffinizing, the sections were digested with proteinase K, DNase and RNase solution. Then hybridization probe was added and heated at 95℃ for 10 minutes to denature the DNA. To allow hybridization of the probe with the target nucleic acid, the sections were incubated at 37℃ overnight. After hybridization finished, the sections were blocked with protein block solution, and combined with the Biotin-Antigen and streptavidine-AP conjugate. In negative controls the hybridization solution (negative control) provided by the Kit was used. HPV staining was documented as either positive or negative.

The differences between the values of different groups were evaluated by the chi-square test. Disease-free survival and overall survival curves were calculated using the Kaplan-Meier method. And the significance of the differences between the curves was estimated by the log-rank test. Multivariate analysis was performed using the Cox logistic regression model by Wald’s backward method. P≤0.05 was considered significant. SPSS 12.0 statistical software was used to conduct all statistical analysis.

The present study was approved by the Ethics Committee of the 2nd Affiliated Hospital, School of Medicine, Zhejiang University.

**Table-I:** Clinicopathological features of patients with LSCC with HPV and PCNA data

		***HPV Infection***			***PCNA Overexpression***		
***Variable***	***Number***	***n***	***%***	***P***	***n***	***%***	***P***
**Sex**							
**Men**	**69**	**29**	**42.0**	**0.10**	**36**	**52.2**	**0.18**
**Women**	**2**	**2**	**100.0**		**2**	**100.0**	
**Age (years)**							
**>60**	**37**	**18**	**48.6**	**0.38**	**21**	**56.8**	**0.57**
**≤60**	**34**	**13**	**38.2**		**17**	**50.0**	
**Tumor site**							
**Glottic**	**39**	**16**	**41.0**	**0.62**	**21**	**53.8**	**0.95**
**Supraglottic**	**32**	**15**	**46.9**		**17**	**53.1**	
**T stage**							
**T1**	**17**	**5**	**29.4**	**0.39**	**8**	**47.1**	**0.32**
**T2**	**17**	**9**	**52.9**		**10**	**58.8**	
**T3**	**23**	**12**	**52.2**		**15**	**65.2**	
**T4**	**14**	**5**	**35.7**		**5**	**35.7**	
**N status**							
**N0**	**50**	**20**	**40.0**	**0.34**	**26**	**52.0**	**0.69**
**N+**	**21**	**11**	**52.4**		**12**	**57.1**	
**Stage**							
**I-II**	**50**	**21**	**42.0**	**0.66**	**27**	**54.0**	**0.90**
**III-IV**	**21**	**10**	**47.6**		**11**	**52.4**	
**Histology grade**							
**G1**	**25**	**9**	**56.0**	**0.43**	**8**	**32.0**	**0.03**
**G2**	**35**	**18**	**45.7**		**23**	**65.7**	
**G3**	**11**	**4**	**45.5**		**7**	**63.6**	

LSCC, Laryngeal squamous cell carcinoma; HPV, Human papillomavirus; PCNA, Proliferating cell nuclear antigen.

**Table-II:** Pair wise correlations between HPV and PCNA expression in 71 LSCC patients

	***HPV***		
	***Normal***	***Overexpression***	***P***
**PCNA**			
**Normal**	**25**	**8**	**0.002**
**Overexpression**	**15**	**23**	

HPV, human papillomavirus; PCNA, proliferating cell nuclear antigen; LSCC, laryngeal squamous cell carcinoma.

**Table-III:** Univariate analysis of prognostic variables for DFS and overall survival in 71 LSCC patients

***DFS***		***Overall survival***		
***Prognostic variable***	***Cumulative 5-year p***		***Cumulative 5-year p***	
	***Survival (%)***		***Survival (%)***	
**Sex**				
**Men**	**69.6**	**0.395**	**74.2**	**0.490**
**Women**	**100.0**		**100.0**	
**Age (years)**				
**>60**	**73.0**	**0.587**	**79.7**	**0.244**
** ≤60**	**67.6**		**69.5**	
**Tumor site**				
**Glottic**	**74.4**	**0.376**	**79.6**	**0.166**
**Supraglottic**	**65.6**		**68.5**	
**T stage**				
**T1 **	**82.4**	**0.114**	**94.1**	**0.019**
**T2 **	**70.6**		**76.5**	
**T3 **	**73.9**		**75.5**	
**T4 **	**50.0**		**50.0**	
**N status**				
**N0 **	**76.0**	**0.083**	**82.2**	**0.015**
**N+ **	**57.1**		**57.1**	
**Stage**				
**I-II **	**78.0**	**0.017**	**84.2**	**0.002**
**III-IV **	**52.4**		**52.4**	
**Histology grade**				
**G1 **	**84.0**	**0.092**	**86.6**	**0.080**
**G2 **	**68.6**		**74.1**	
**G3**	**45.5**		**51.9**	
**HPV**				
**normal**	**60.0**	**0.029**	**66.2**	**0.064**
**overexpression**	**83.9**		**86.6**	
**PCNA**				
**Normal**	**75.8**	**0.369**	**81.6**	**0.244**
**overexpression**	**65.8**		**66.6**	

DFS, disease-free survival; LSCC, laryngeal squamous cell carcinoma; HPV, human papillomavirus; PCNA, proliferating cell nuclear antigen.

## RESULTS

 The clinicopathological features of the patients are summarized in Table-I. There were 69 men and 2 women, with a mean age of 61.6 ± 9.6 years (rang 43-80 years). Patients were followed for a median of 44 months (rang 6-123 months). Among the 71 cases, 31 (43.7%) showed infection of HPV and 38 (53.5%) showed overexpression of PCNA. No significant difference of HPV infection in clinicopathological features was detected. The differences of PCNA expression in clinicopathological features were also not significant except histology grade (p=0.03). However, HPV and PCNA stainings were associated with each other (Table-II). HPV infection was correlated with PCNA overexpression (p=0.002).

***Disease-free survival:*** During the follow-up, 20 patients relapsed. Among these patients, 9 recurred at the primary site, 9 recurred in the neck and two patients had distant metastasis. In univariate analyses, stage (p=0.017) and HPV infection (p=0.029) were the statistically significant predictors of DFS (Table-III) and HPV infection is a favorable predictor of DFS. The expression of PCNA (p=0.369) is not a significant predictor of DFS (Table-III). For multivariate analyses, only the variables significant in univariate analyses were included, and HPV infection (p=0.02) and stage (p=0.01) were significant factors.

***Overall survival:*** Fifteen patients died from the disease during the follow-up period. In univariate analyses, significant predictors of overall survival were T stage (p=0.019), N status (p=0.015) and stage (p=0.002) (Table-III). While HPV infection (p=0.064) showed a trend as a significant prognostic factor and is a favorable predictor. The expression of PCNA (p=0.244) is also not a significant predictor of overall survival (Table-II). In multivariate analyses, only the variables significant in univariate analyses were included. Only stage (p=0.004) was statistically significant predictor of overall survival.

## DISCUSSION

Recently, many studies have focused on the relationship between HPV and LSCC. In the present study, we found that HPV infection is more frequent in elderly patients than younger patients, and more frequent in T2-T4 stage than T1 stage, and detected more frequently in patients with nodal metastasis. However, none of these differences is statistically significant. Morshed^10^ also detected HPV infection in LSCC. Similar to our results, they indicated that no significant correlation was found between the incidence of HPV and the epidemiological and histological grade, and clinical stage of tumors. In their results, HPV is also detected more frequently in T2-T4 stage and in patients with nodal relapse, but more HPV-positive patients are younger.

Moreover, same as our results, no obvious difference of HPV infection between supraglottic and glottic tumor was found. Jacob et al^11^ explored the presence of HPV in LSCC and found that there was no significant correlation between the presence of HPV in laryngeal carcinomas and clinicopathological variables such as age, site, grade, lymph node status, and stage of the disease. While Hoshikawa et al^12^ showed that laryngeal carcinomas of glottic origin had higher HPV positive rates (4 of 9 cases, 44.4%) than those of other sites. Nichols et al^13^ detected HPV-16 infection in oropharyngeal squamous cell carcinoma and demonstrated that HPV-16 infected tumors are correlated with smaller primary site size. Although the relationships between HPV infection and clinical characteristics of patients with LSCC have been explored in multiple studies, the results are still controversial.

HPV infection is considered as an indicator of better prognosis in patients with HNSCC in many studies. Pintos et al^14^ reported that HPV positive HNSCCs have higher survival rates than HPV negative cases. Fakhry et al^3^ evaluated the association of HPV status with therapeutic response and survival in patients with head and neck squamous cell carcinomas, and demonstrated that patients with HPV positive tumors had higher response rates after chemoradiation treatment and significantly improved overall survival. Ang KK et al^2^ analysed the association between tumor HPV status and survival among patients with stage III or IV oropharyngeal squamous cell carcinoma and found HPV status is a strong and independent prognostic factor. In present study, the data indicated that the HPV infection is a favorable predictor of either disease-free survival or overall survival of patients with LSCC. However, there are still controversial and contradictory results. Stephen et al^15^ detected HPV in 79 patients with primary LSCC and indicated that no differences in survival between HPV positive and HPV negative patients is found. Morshed^10^ investigated HPV status in patients with LSCC, and found that HPV positive laryngeal cancer patients had no significantly better overall survival and disease specific survival when compared with patients with HPV negative tumors.

PCNA is an E2F-regulated gene product which is induced in papillomas by the presence of E7.^16^^,^^17^ E7, which is a viral oncoprotein, can be produced by HPV, and binds to pRb leading to releasing the E2F transcription factor which can activate PCNA gene. Thus, PCNA can be regarded as a surrogate marker of viral early gene activity in papillomavirus infected tissue.^18^ Branca et al^19^ observed that in patients with cervical intraepithelial neoplasia (CIN) or cervical carcinomas PCNA expression intensity was significantly associated with the detection of high-risk HPV in the lesions, being significantly more intense in high-risk HPV positive lesions. Similar correlation between HPV and PCNA was also found by Noya et al,^20^ who showed that HPV E7 protein induces PCNA protein in differentiated keratinocytes by reactivating the transcription of the PCNA.

While other studies failed to establish any direct association between the PCNA expression and HPV infection. Wang et al^9^ evaluated PCNA expression and HPV status in patients with cervical cancer and no significant correlation was found between these two. To our knowledge, there has been little study to report PCNA expression and HPV presence in the patients with LSCC. Jacob et al^11^ used PCR to detect the infection of HPV and immunohistochemistry to detect the PCNA expression in 41 patients with LSCC. Their results showed that HPV infection was correlated with increased expression of PCNA. However, they did not explore the role of HPV infection in prognosis of patients with LSCC.

Moreover, although PCR is more sensitive than other approaches, as total DNA are extracted from tissue samples, so the detected HPV DNA can be derived from non-cancerous cells, a tumor surface contamination or just only few cancer cells.^21^ Compared with PCR method, ISH can identify the HPV infection in the tumor cell nuclei, which could provide more reliable results. In this study, we used in situ hybridization to explore HPV infection and more patients were included. The result showed that HPV infection was associated with PCNA overexpression. Moreover, in present study, there was a significant difference of PCNA expression in histology grade but no significant difference of PCNA expression in other clinicopathological features could be detected, and the expression of PCNA is not a significant predictor of disease-free survival or overall survival in LSCC patients. Similar results were also demonstrated by other studies.^22^

## CONCLUSION

Either HPV or PCNA has been investigated as a LSCC tumor marker in many studies. However, the relationship between HPV infection and PCNA expression in LSCC tumors has not been well documented. The present study showed that HPV infection is a favorable prognostic factor in patients with LSCC although no significant difference of HPV infection in the clinicopathological features was detected. Furthermore, the infection of HPV is significantly correlated with PCNA overexpression in LSCC patients.

## Notes

***Source of Funding:*** None.

## References

[1] Syrjanen KJ, Surjanen SM (1981). Histological evidence for the presence of condylomatous epithelial lesions in association with laryngeal squamous cell carcinoma. J Otorhinolaryngol Relat Spec.

[2] Ang KK, Harris J, Wheeler R, Weber R, Rosenthal DI, Nquyen-Tan PF (2010). Human papillomavirus and survival of patients with oropharyngeal cancer. N Engl J Med..

[3] Fakhry C, Westra WH, Li S, Cmelak A, Ridge JA, Pinto H (2008). Improved survival of patients with human papillomavirus-positive head and neck squamous cell carcinoma in a prospective clinical trial. J Natl Cancer Inst.

[4] Adelstein DJ, Ridge JA, Gillison ML, Chaturvedi AK, D'Souza G, Gravitt PE (2009). Head and neck squamous cell cancer and the human papillomavirus: summary of a National Cancer Institute State of the Science Meeting, November 9-10, 2008, Washington, D. C. Head Neck.

[5] Diez M, Ramos P, Medrano MJ, Muguerza JM, Villeta R, Lozano O (2003). Preoperatively irradiated rectal carcinoma: analysis of the histopathologic response and predictive value of proliferating cell nuclear antigen immunostaining. Oncology.

[6] Buyukbayram H, Cureoglu S, Arslan A, Isikakdogan AR (2004). Prognostic value of PCNA and mutant p53 expression in laryngeal squamous cell carcinoma. Cancer Invest.

[7] Melendy T, Sedman J, Stenlund A (1995). Cellular factors required for papillomavirus DNA replication. J Virol.

[8] Middleton K, Peh W, Southern S, Griffin H, Sotlar K, Nakahara T (2003). Organization of human papillomavirus productive cycle during neoplastic progression provides a basis for selection of diagnostic markers. J Virol.

[9] Wang JL, Zheng BY, Li XD, Angstrom T, Lindstrom MS, Wallin KL (2004). Predictive significance of the alterations of p16INK4A, p14ARF, p53, and proliferating cell nuclear antigen expression in the progression of cervical cancer. Clin Cancer Res..

[10] Morshed K (2010). Association between human papillomavirus infection and laryngeal squamous cell carcinoma. J Med Virol.

[11] Jacob SE, Sreevidya S, Chacko E, Pillai MR (2002). Cellular manifestations of human papillomavirus infection in laryngeal tissues. J Surg Oncol.

[12] Hoshikawa T, Nakajima T, Uhara H, Gotoh M, Shimosato Y, Tsutsumi K (1990). Detection of human papillomavirus DNA in laryngeal squamous cell carcinomas by polymerase chain reaction. Laryngoscope.

[13] Nichols AC, Faquin WC, Westra WH, Mroz EA, Bequm S, Clark JR (2009). HPV-16 infection predicts treatment outcome in oropharyngeal squamous cell carcinoma. Otolaryngol Head Neck Surg.

[14] Pintos J, Franco EL, Black MJ, Bergeron J, Arella M (1999). Human papillomavirus and prognoses of patients with cancers of the upper aerodigestive tract. Cancer.

[15] Stephen JK, Chen KM, Shah V, Havard S, Lu M, Schweitzer VP (2012). Human papillomavirus outcomes in an access-to-care laryngeal cancer cohort. Otolaryngol Head Neck Surg.

[16] Tommasi S, Pfeifer GP (1999). In vivo structure of two divergent promoters at the human PCNA locus. Synthesis of antisense RNA and S phase-dependent binding of E2F complexes in intron 1. J Biol Chem.

[17] Klaes R, Benner A, Friedrich T, Ridder R, Herrington S, Jenkins D (2002). p16INK4a immunohistochemistry improves interobserver agreement in the diagnosis of cervical intraepithelial neoplasia. Am J Surg Pathol.

[18] Middleton K, Peh W, Southern S, Griffin H, Sotlar K, Nakahara T (2003). Organization of human papillomavirus productive cycle during neoplastic progression provides a basis for selection of diagnostic markers. J Virol.

[19] Branca M, Ciotti M, Giorgi C, Santini D, Di Bonito L, Costa S (2007). Up-regulation of proliferating cell nuclear antigen (PCNA) is closely associated with high-risk human papillomavirus (HPV) and progression of cervical intraepithelial neoplasia (CIN), but does not predict disease outcome in cervical cancer. Eur J Obstet Gynecol Reprod Biol.

[20] Noya F, Chien WM, Wu X, Banerjee NS, Kappes JC, Broker TR (2002). The promoter of the human proliferating cell nuclear antigen gene is not sufficient for cell cycle-dependent regulation in organotypic cultures of keratinocytes. J Biol Chem.

[21] Ambretti S, Venturoli S, Mirasoli M, La Placa M, Bonvicini F, Cricca M (2007). Assessment of the presence of mucosal human papillomaviruses in malignant melanomas using combined fluorescent in situ hybridization and chemiluminescent immunohistochemistry. Br J Dermatol.

[22] Jiang H, Yang BB (2009). p53, epidermal growth factor receptor and proliferating cell nuclear antigen in laryngeal squamous cell carcinoma are not predictive markers for the effect of adjuvant radiotherapy. Acta Otolaryngol.

